# Differential Association of Inflammation With Pain and Physical Function in Knee Osteoarthritis by Race Focusing on Non-Hispanic Whites and Asian Americans: Pilot Study in Florida

**DOI:** 10.2196/75086

**Published:** 2026-01-13

**Authors:** Chiyoung Lee, C Kent Kwoh, Hyochol Ahn

**Affiliations:** 1 College of Nursing, University of Arizona, 1305 N Martin Avenue, Tucson, AZ, 85721-0203, United States, 1 9842096277; 2 College of Medicine, University of Arizona, Tucson, AZ, United States

**Keywords:** Asian Americans, inflammation, knee osteoarthritis, pain, physical function

## Abstract

**Background:**

The current body of work has not yet addressed the potential racial differences in the relationship between systemic inflammation and knee osteoarthritis (OA) symptoms, including pain and physical function.

**Objective:**

This pilot study aimed to investigate this association specifically among non-Hispanic Whites and Asian Americans.

**Methods:**

We cross-sectionally analyzed 40 community-dwelling participants aged 50‐70 years with self-reported knee OA pain, including 20 non-Hispanic Whites and 20 Asian Americans. Knee OA symptoms were assessed using the Western Ontario and McMaster Universities Osteoarthritis Index (WOMAC) pain and physical function subscales. The serum levels of C-reactive protein (CRP), tumor necrosis factor-alpha (TNF-α), and interleukin-10, as systemic inflammatory markers, were measured. Univariate and multivariable analyses, using stepwise linear regression models, were conducted to examine the correlation between these inflammatory markers and OA symptoms, with systematic adjustment for age.

**Results:**

In non-Hispanic Whites, the above inflammatory markers did not correlate with knee pain or physical function. In Asian Americans, bivariate analyses revealed that CRP and TNF-α levels were associated with worse WOMAC pain scores (*r*=1.325, *P*=.041; and *r*=2.418, *P*=.036, respectively), and CRP levels were also linked to worse WOMAC physical function scores (*r*=4.950, *P*=.035). Multivariate analyses confirmed the association of CRP levels with both worse WOMAC pain (*β*=1.328, *P*=.046) and physical function (*β*=4.974, *P*=.034) scores in Asian Americans.

**Conclusions:**

CRP may be a clinically relevant marker for knee OA symptoms, specifically in Asian Americans; however, caution is warranted owing to the exploratory nature of this study. Future research is set to benefit from leveraging a larger sample, incorporating additional inflammatory markers, and including racially diverse samples to validate and augment these findings.

## Introduction

### Background

Knee osteoarthritis (OA) is one of the leading causes of pain, daily living impairments, and disability in people aged ≥45 years. Although local inflammation aggravates OA joint pathologies, systemic inflammatory markers are also elevated in OA, especially in the presence of symptoms such as pain and reduced physical function [1,2]. Notwithstanding, the evidence remains controversial. The pooled results of a meta-analysis indicated a weak correlation between serum C-reactive protein (CRP) levels and knee OA pain [1]. Other studies have identified no significant association between serum CRP levels and knee OA pain or physical function [3,4]. A recent systematic review also reported conflicting associations of other markers, such as interleukin (IL)-6, with pain scores in patients with knee OA [5].

Several researchers argue that inconsistent evidence regarding the relationship between inflammation and knee OA symptoms is attributable to sex-specific differences [6-8]. However, considerably less attention has been focused on the possibility of racial/ethnic disparities in these relationships. Racial/ethnic minority groups, owing to systemic inequities and environmental challenges, may develop a different inflammatory fingerprint than their non-Hispanic White counterparts (ie, epigenetics) [9]. They are also disproportionately susceptible to chronic knee OA pain [10-12]. Nonetheless, most studies on inflammation and knee OA symptoms have failed to specify the racial composition of their samples, predominantly included non-Hispanic White participants, or adopted typical approaches that report average effects from race/ethnicity-adjusted analyses, possibly obscuring crucial dissimilarities. Overlooking such differences potentially hinders the development of personalized approaches to analgesic care and interventions that improve physical function, ultimately impeding efforts to reduce pain inequities across groups.

To our knowledge, no study has elucidated the potential racial/ethnic differences in the relationship between systemic inflammatory markers and knee OA symptoms. Only recently, Overstreet et al [13] found that the expressions/profiles of biomarkers underlying inflammation associated with chronic low back pain-related outcomes (ie, pain interference, pain at rest, and movement-evoked pain) differed between non-Hispanic Whites and non-Hispanic Blacks.

### Study Aim

This pilot study investigated the relationship between inflammation and knee OA symptoms (ie, pain and physical function) by race. Specifically, we compared non-Hispanic Whites to Asian Americans. Asian Americans constitute a rapidly growing minority yet have been underrepresented in knee OA research, despite emerging evidence indicating that they experience greater knee OA symptoms than non-Hispanic Whites [10,11]. This underscores the critical importance of studying this population.

## Methods

### Study Design and Participants

This cross-sectional analysis used baseline data from a randomized controlled trial (RCT) registered at clinicaltrials.gov (NCT02512393) to assess the efficacy of transcranial direct current stimulation (tDCS) in mitigating knee OA pain. The parent trial employed a double-blind, sham-controlled, parallel-group design, in which participants were randomly assigned to receive either active tDCS (n=20) or sham tDCS (n=20). Participants underwent daily 20-minute stimulation sessions for 5 consecutive days. Additional details regarding the design and procedures of the parent RCT are reported elsewhere [14,15]. At baseline, a total of 40 individuals with self-reported knee OA pain (20 non-Hispanic Whites and 20 Asian Americans) were recruited in North Central Florida between September 2015 and August 2016. Prior to the intervention, participants completed comprehensive baseline assessments, including demographic characteristics, clinical measures, knee OA pain severity, physical function, and relevant biological factors. The breadth and depth of these baseline data provided a robust foundation for the present cross-sectional analyses.

Recruitment was conducted through a combination of posted flyers, email advertisements, and community-based outreach efforts at local clinics, hospital-based outpatient services, and community centers. Flyers and electronic announcements described the study purpose, eligibility criteria, and contact information for the research team. Interested individuals contacted the study staff and were provided with additional information about the study. Some participants were recruited via direct referral from treating clinicians in outpatient settings. Potential participants then underwent a standardized screening process, which included an initial telephone screening followed by an in-person eligibility assessment to confirm the inclusion and exclusion criteria prior to enrollment.

Because the present analyses were conducted using baseline data from a parent RCT, the eligibility criteria reflected those of the parent trial rather than being tailored specifically for the current secondary analysis. Participants were eligible if they were aged 50‐70 years; reported unilateral or bilateral knee OA pain according to the American College of Rheumatology criteria [16,17]; were able to speak and read English; were willing to be randomly assigned to either the intervention or control group; were available to complete five consecutive daily tDCS sessions and weekly follow-up phone assessments for 3 weeks; had no plans to change pain-related medication regimens during the study period; had no contraindications identified through the tDCS safety screening questionnaire (eg, epilepsy) [18]; and were willing and able to provide written informed consent prior to enrollment. Exclusion criteria ensured that participants did not have concurrent medical conditions that could confound OA-related outcomes or coexisting diseases that could hinder protocol completion. Thus, the following were the exclusion criteria: (1) having undergone prosthetic knee replacement or non-arthroscopic surgery on the affected knee, (2) a serious medical illness, such as uncontrolled hypertension, heart failure, or a recent history of acute myocardial infarction, (3) peripheral neuropathy, (4) systemic rheumatic disorders, such as rheumatoid arthritis, systemic lupus erythematosus, and fibromyalgia, (5) alcohol or substance abuse, (6) cognitive impairment, defined as a Mini-Mental Status Exam score of 23 or lower, (7) a history of brain surgery, tumor, seizure, stroke, or intracranial metal implantation, (8) pregnancy or lactation, and (9) hospitalization for a psychiatric illness within the past year.

### Measurement

The collected basic characteristics included age, sex (male vs female), marital status (partnered vs unpartnered), BMI (kg/m²), and Kellgren–Lawrence (KL) radiographic grade (0‐1 vs 2‐4), pain catastrophizing, and negative affect. The study assessed pain catastrophizing using the pain catastrophizing scale (PCS), a 13-item measure designed to evaluate catastrophic thinking related to pain across three dimensions: rumination, magnification, and helplessness [19,20]. Each item was scored on a 5-point Likert scale ranging from “not at all” (0) to “all the time” (4). The PCS demonstrated adequate internal consistency, with subscale alphas ranging from 0.66 to 0.87 (α for all items=0.87) [19], and its sensitivity to psychosocial interventions for chronic pain has been well established [21]. Negative affect was assessed using the 10-item negative affect subscale of the Positive and Negative Affect Schedule (PANAS-NA) [22]. Respondents are asked to rate on a 5-point scale (1 = “very slightly or not at all”; 5 = “extremely”) their agreement with 10 descriptors of negative affect (afraid, ashamed, distressed, guilty, hostile, irritable, jittery, nervous, scared, and upset). The PANAS has been validated and demonstrates reliability, with an alpha coefficient range of .84 to .87 for negative affect [23].

### Knee OA Symptoms: Knee OA Pain and Physical Function

Knee OA pain and physical function were measured using the Western Ontario and McMaster Universities Osteoarthritis Index (WOMAC) pain and physical function subscales, where higher scores indicate greater pain and physical functional disability [24]. The pain subscale includes 5 items on a 5-point Likert scale (0 being none to 4 being extreme) measuring the pain severity during walking, climbing stairs, sleeping, resting, and standing. The participants’ responses to the pain questions were summed up to derive an aggregated score of pain intensity (range 0‐20). The physical function subscale asks patients to rate the degree of difficulty in accomplishing 17 activities of daily living on a 5-point scale (0 being none to 4 being extreme). The participants’ responses were aggregated to produce a composite score of functional disability (range 0‐68). The subscales in WOMAC demonstrate reliability and validity in evaluating knee OA in patients [24,25].

### Inflammatory Markers

In this study, we also gathered data regarding the following inflammatory markers: CRP, tumor necrosis factor-alpha (TNF-α), IL-1β, IL-6, and IL-10. Owing to substantial missing data (45.0%‐80.0%), we excluded IL-1β and IL-6 from the current analysis. In the original study [15], blood samples were obtained prior to treatment initiation (ie, tDCS) on day 1 and after completing the fifth treatment on day 5. For our analysis, we utilized pre-treatment data acquired on day 1. Blood was drawn into ethylenediaminetetraacetic acid plasma tubes. Samples were inverted five times and stored on ice until further processing. Within 30 min of being collected, samples were centrifuged at 1600×*g* and 4°C for 15 min, aliquoted, and immediately stored in a −80°C freezer.

The plasma samples underwent solid-phase extraction using an Oasis™ Hydrophilic–Lipophilic-Balanced (30 mg) 96-well plate along with a vacuum manifold (Waters Corp.), according to the manufacturer’s protocol. Briefly, the plate was conditioned with acetonitrile and equilibrated twice with 0.1% trifluoroacetic acid (TFA) in high-performance liquid chromatography (HPLC)-grade water. Samples were acidified with 1% TFA (1:1) and loaded onto the plate. The plate was washed thrice with 0.1% TFA in HPLC-grade water. The samples were eluted in 60% acetonitrile/40% HPLC-grade water/0.1% TFA and dried in a Savant AES1010 Automatic Environmental SpeedVAC® w/VaporNet Radiant Cover (Thermo Fisher Scientific). Thereafter, they were reconstituted using the original sample volume in assay buffer.

Plasma CRP levels were measured in duplicate using enzyme-linked immunosorbent assays, following the manufacturers’ instructions (cat# DCRP00, R&D Systems, Minneapolis, MN; cat# ADI-900‐071, Enzo Life Sciences, Inc., Farmingdale, NY, respectively). For CRP, the average intra- and interassay CV values were <10.0% and <7.0%, respectively. TNF-α and IL-10 plasma levels were measured in triplicate using a commercial multiplex immunoassay kit (cat# HCYTMAG-60Kl; MilliporeSigma, Burlington, MA) and analyzed using the MILLIPLEX® Analyzer 3.1 xPONENT® System (Luminex Corporation) . Data acquisition was accomplished using the same system and data analysis performed via MILLIPLEX® Analyst Software. Intra- and interassay CVs were <19.0% for all markers.

### Statistical Analysis

Descriptive and comparative statistics were employed to determine sample characteristics. As the inflammatory markers were not normally distributed, log transformation was applied to mitigate skewness. When missing data were present (CRP, n=4), listwise deletion was performed, resulting in a streamlined dataset for analysis. Race-stratified analyses were conducted owing to an observed interaction between race and certain inflammatory markers, such as CRP (data not shown). We examined the relationships between BMI and each inflammatory marker, aiming to circumvent possible collinearity (since adiposity proves to be significantly associated with systemic inflammation), and specifically investigated their associations with knee OA symptoms. Multivariable linear regression models were used in the main analysis of each outcome.

Explanatory variables included age, sex, marital status, BMI, and KL radiographic grade, all of which potentially affect both inflammation and knee OA symptoms [26,27]. Pain catastrophizing and negative affect were also considered because of their relation to knee OA symptoms [28-30]. Candidate variables comprised those with *P*<.200 in bivariate analyses: single-factor analysis of variance in cases of variance equality, the Kruskal–Wallis test for qualitative variables, and the simple linear regression test for quantitative variables. Multivariable analysis based on stepwise selection at an alpha value of .05 was conducted to preserve the most relevant variables in the model and distinguish those independently associated with the outcomes. In all multivariable models, systematic adjustment for age was performed. Finally, we conducted diagnostic tests to ensure that the multivariable models satisfied linear regression model assumptions. All statistical analysis was carried out using R Studio (version 4.0.2; R Foundation for Statistical Computing) [31].

### Ethical Considerations

The Institutional Review Board (IRB) of the University of Arizona considers investigators engaged in research if (1) they interact with participants for research purposes; (2) they have access to identifying study information; (3) they obtain informed consent from research participants; or (4) the University of Arizona directly receives part of federal funds for the study (ie, the University of Arizona is the prime awardee). If none of the above are true, then the researchers would not require IRB approval. Thus, this secondary analysis of deidentified data from an existing RCT was determined to be exempt from IRB review. Informed written consent was obtained from all participants in the parent trial, and participants were compensated for their time and participation.

## Results

Table 1 presents the characteristics of the participants by race. The groups differed in terms of age, BMI, KL radiographic grade, and pain catastrophizing. Asian American participants were significantly younger than non-Hispanic White participants (mean [SD] 54.80 [7.74] vs 65.10 [7.41] years; *P*=.001) and had a lower BMI (mean [SD] 25.02 [3.59] vs 27.98 [3.28] kg/m²; *P*=.001). A greater proportion of Asian Americans had lower KL radiographic grades (0‐1) compared with non-Hispanic Whites (80% vs 25%; *P*=.001). Asian American participants also reported higher pain catastrophizing scores than non-Hispanic White participants (mean [SD] 1.33 [1.25] vs. 0.31 [0.74]; *P*=.004). There were no significant differences between the groups in the levels of CRP, TNF-α, and IL-10, or in any WOMAC subscales (*P*>.05).

**Table 1.** Comparison of basic characteristics, inflammatory markers, and WOMAC^a^ subscale scores between non-Hispanic Whites and Asian Americans (n=40).

Variables	Non-Hispanic Whites (n=20)	Asian Americans (n=20)	*P* value
Age (years), mean (SD)	65.10 (7.41)	54.80 (7.74)	.001^b^
Sex, n (%)			.205
Male	12 (60)	7 (35)	
Female	8 (40)	13 (65)	
Marital status			.127^c^
Married/partnered	13 (65)	18 (90)	
Nonmarried/unpartnered	7 (35)	2 (10)	
Body mass index, kg/m^2,^ mean (SD)	27.98 (3.28)	25.02 (3.59)	.001^b^
Kellgren-Lawrence radiographic grade (grade 0‐1)			.001^c^^,^ ^b^
Grade 0‐1	5 (25)	16 (80)	
Grade 2‐4	15 (75)	4 (20)	
Pain catastrophizing scale score, range: 0‐6; mean (SD)	0.31 (0.74)	1.33 (1.25)	.004^b^
Negative affect scale score, range: 10‐50, mean (SD)	14.00 (4.29)	20.15 (9.02)	.165
Inflammatory markers, mean (SD)			
C-reactive protein, ng/nl	2295.27 (3002.13)	1001.12 (898.91)	.114
Tumor necrosis factor-α, pg/ml	10.33 (5.56)	7.51 (3.67)	.067
Interleukin-10, pg/ml	8.38 (5.27)	6.78 (3.69)	.273
Clinical pain measures, mean (SD)			
WOMAC pain, range: 0‐20	4.90 (2.61)	4.40 (2.74)	.559
WOMAC physical function, range: 0‐68	16.50 (9.86)	13.6 (9.95)	.361

a WOMAC: Western Ontario and McMaster Universities Osteoarthritis.

b Significant results.

c Fischer exact test.

Table 2 presents the relationships between BMI and inflammatory markers by race. Pearson correlation analyses showed no statistically significant associations between BMI and log-transformed CRP, TNF-α, or IL-10 in either non-Hispanic Whites or Asian Americans (all *P*>.05). Similarly, ANOVA analyses revealed no significant differences in inflammatory marker levels across BMI categories in either group. These findings indicate that BMI was not strongly correlated with inflammatory markers in this sample and did not raise major concerns about multicollinearity in subsequent analyses, as shown in Table 2.

**Table 2.** Relationships between BMI and inflammatory markers by race (n=40).

	Non-Hispanic Whites (n=20)			Asian Americans (n=20)		
	log(CRP)^a^, ng/nl	log(TNF-α)^b^, pg/ml	log(IL-10)^c^, pg/ml	log(CRP), ng/nl	log(TNF-α), pg/ml	log(IL-10), pg/ml
Pearson correlation coefficient	0.179	−0.079	−0.190	0.364	0.292	0.218
*P* value	.507	.740	.421	.115	.212	.356
18.5≤BMI<25^d^, mean (SD)	7.49 (1.53)	2.22 (0.59)	2.01 (0.71)	6.13 (0.91)	1.79 (0.62)	1.74 (0.64)
25≤BMI<30^e^, mean (SD)	6.75 (0.54)	2.20 (0.33)	1.97 (0.71)	7.11 (0.79)	1.97 (0.38)	1.74 (0.33)
BMI≥30^f^, mean (SD)	7.73 (0.73)	2.26 (0.64)	1.87 (0.82)	6.71 (1.07)	2.22 (0.57)	2.11 (0.67)
ANOVA *P* value	.194	.971	.929	.096	.551	—^g^

a CRP: C-reactive protein.

b TNF-α: tumor necrosis factor-alpha.

c IL-10: interleukin-10.

d Non-Hispanic Whites (n=5), Asian Americans (n=11).

e Non-Hispanic Whites (n=9), Asian Americans (n=7).

f Non-Hispanic Whites (n=6), Asian Americans (n=2).

g not applicable.

Table 3 presents the results of bivariate and multivariable analyses in both non-Hispanic Whites and Asian Americans. In non-Hispanic Whites, both analyses indicated that only pain catastrophizing was associated with worse WOMAC pain score and WOMAC physical function score (*P*<.050). In Asian Americans, bivariate analysis indicated that CRP and TNF-α levels were associated with a worse WOMAC pain score (*r*=1.325, *P*=.041; *r*=2.418, *P*=.036, respectively), while the CRP level was also related to a worse WOMAC physical function score (*r*=4.950, *P*=.035). In multivariable analysis adjusting for age, only the CRP level was associated with a worse WOMAC pain score (*β*=1.328, *P*=.046) and a WOMAC physical function score (*β*=4.974, *P*=.034) in Asian Americans. Of note, the IL-10 level was not significantly associated with WOMAC pain or physical function scores in either non-Hispanic White or Asian American participants in bivariate or multivariable analyses.

**Table 3.** Bivariate and multivariable analyses (n=40).

	Non-Hispanic Whites (n=20)								Asian Americans (n=20)							
	WOMAC^a^ pain				WOMAC physical function				WOMAC pain				WOMAC physical function			
	Bivariate analysis		Multivariable analysis		Bivariate analysis		Multivariable analysis		Bivariate analysis		Multivariable analysis		Bivariate analysis		Multivariable analysis	
	*r*	*P* value	*β* (SE)^b^	*P* value	*r*	*P* value	*β* (SE)	*P* value	*r*	*P* value	*β* (SE)	*P* value	*r*	*P* value	*β* (SE)	*P* value
Age	−0.133	.101	−.062 (0.058)	.304	−0.170	.592	.048 (0.283)	.868	0.030	.724	.032 (0.076)	.683	0.278	.360	.284 (0.266)	.300
Sex	0.043	.378	—^c^	—	0.108	.157	—	—	0.027	.488	—	—	0.001	.921	—	—
Marital status	0.037	.414	—	—	0.004	.802	—	—	0.013	.637	—	—	0.000	.931	—	—
BMI	0.211	.259	—	—	0.682	.335	—	—	0.029	.872	—	—	−0.144	.828	—	—
KL^d^ radiographic grade	0.087	.207	—	—	0.002	.860	—	—	0.006	.754	—	—	0.001	.897	—	—
Pain catastrophizing	2.548	<.001^e^	2.370 (0.572)	.001^e^	7.115	.013^e^	7.252 (2.764)	.018^e^	0.478	.345	—	—	1.395	.449	—	—
Negative affect	−0.068	.631	—	—	−0.720	.167	—	—	0.130	.053	—	—	0.279	.269	—	—
log(CRP)^f^, ng/nl	−1.035	.157	—	—	−2.789	.283	—	—	1.325	.041^e^	1.328 (0.616)	.046^e^	4.950	.035^e^	4.974 (2.157)	.034^e^
log(TNF-α)^g^, pg/ml	−0.115	.930	—	—	−2.613	.596	—	—	2.418	.036^e^	—	—	6.716	.118	—	—
log(IL-10)^h^, pg/ml	−0.861	.395	—	—	−4.158	.273	—	—	1.134	.349	—	—	2.297	.604	—	—

a WOMAC: Western Ontario and McMaster Universities Osteoarthritis.

b SE: standard error.

c not applicable.

d KL: Kellgren-Lawrence.

e Significant results.

f CRP: C-reactive protein.

g TNF-α: tumor necrosis factor-alpha.

h IL-10: interleukin-10.

## Discussion

### Principal Findings and Comparison With Previous Works

This pilot study investigated whether differences in the relationship between inflammation and knee OA symptoms (pain and physical function) exist between non-Hispanic Whites and Asian Americans. Our adjusted analyses indicated that the CRP level persists as a clinically relevant marker for both knee OA pain and functional disability in Asian Americans. The findings emphasize that inflammatory underpinnings of knee OA symptoms potentially vary among specific racial groups, echoing the results of Overstreet et al [13], which focused on patients with chronic low back pain.

However, interpretation needs caution. The proportion of individuals with early-stage knee OA (KL radiographic grade 0‐1) significantly differed between the two groups, with 25% and 80% of non-Hispanic Whites and Asian Americans falling into this category, respectively. Evidence suggests that in early-stage knee OA, inflammation is a major reason why patients seek medical assistance in outpatient departments, and anti-inflammatory treatment may be more effective during this stage; in contrast, in late-stage knee OA, pain may not primarily originate from inflammation but rather from other sources that require further investigation [32], a phenomenon corroborated by our study. Furthermore, in the current study, men constituted 60% and 35% of the non-Hispanic White and Asian American samples, respectively. Although this sex composition was not statistically significant, a previous study has reported sex-specific relationships exhibiting weaker associations of CRP and TNF-α with knee pain among men [7]; this possibly, in part, explains our insignificant findings for non-Hispanic Whites.

The findings wherein no relationships were established between inflammatory markers and knee OA symptoms among non-Hispanic Whites aligns with an earlier study predominantly based on non-Hispanic White samples [3]. However, it contrasts with results reported by Zhu et al [33], who found serum CRP to be cross-sectionally and longitudinally associated with knee pain in patients with knee OA, as well as with other research reporting significant associations between knee OA pain and TNF-α [7,34,35]. Nonetheless, direct comparisons are challenging owing to the unknown racial composition of these studies, our pilot study’s cross-sectional nature characterized by small sample sizes, varying socioeconomic and clinical characteristics across the study populations, and the multidimensional nature of pain assessed using various tools across the studies.

Notably, the IL-10 level was not associated with knee OA symptoms in either non-Hispanic White or Asian American participants. IL-10 is an anti-inflammatory cytokine that regulates immune homeostasis and may slow the progression of knee OA [36]. Several studies in knee OA have reported null or negative associations between IL-10 levels and clinical pain and function [7,37,38]. Imamura et al [37] found no relationship between serum IL-10 levels and WOMAC pain scores in individuals with painful knee OA and sensitization. Perruccio et al [7] reported that higher serum IL-10 levels were associated with lower WOMAC pain scores, regardless of sex. Similarly, Zhu et al [38] examined longitudinal associations between inflammatory and metabolic markers and WOMAC outcomes and found that an IL-10–related component, characterized by predominantly anti-inflammatory markers, was negatively associated with WOMAC pain and function scores. Yet, none of the aforementioned studies examined racial differences in the associations between IL-10 and knee OA symptoms, limiting comparisons with the present findings and underscoring the need for future research that explicitly considers race.

As health care practices in the United States shift toward precision and targeted medicine, considering demographic factors that potentially influence mechanistic processes is imperative [13]. We acknowledge that our pilot investigation may not have immediate clinical implications. However, our race-specific findings may inform health care providers that treatments chiefly developed based on data from non-Hispanic Whites may not provide optimal analgesic care for Asian Americans and suggest that the future development of novel OA treatment approaches may ultimately vary according to race, depending on the therapeutic target.

Based on our findings, future studies involving larger samples are required to validate our results and facilitate more advanced modeling (eg, with mediators/moderators) to augment current knowledge. In addition, future studies may substantially benefit from leveraging a larger pool of biomarkers that could be analyzed as possible correlational factors for knee OA symptoms. Simultaneously, several studies [5,39], including our own, have relied on a single marker as a measure of inflammatory status; however, recognizing that the inflammatory system is complex and involves multiple feedback mechanisms is indispensable. For example, a study by Zhu et al [38] attempted to address these issues by examining the patterns of 19 different inflammatory markers and adipokines derived from principal component analysis and subsequently exploring their association with knee OA symptoms. Future studies should ascertain whether these associations differ across racial/ethnic groups and also include individuals from other racial/ethnic minority groups, such as non-Hispanic Blacks and Hispanics.

### Limitations

This pilot study has certain limitations. First, as this study represents a secondary, cross-sectional analysis of baseline data from a parent RCT, both the study design and the measures available for analysis were determined by the objectives of the parent trial rather than the specific aims of the current study. In addition, the sample size was defined by the feasibility-oriented goals of the parent pilot trial, rather than by statistical power considerations for the present secondary analysis. As such, the findings should be interpreted as exploratory. These design features may also introduce potential sources of bias, including selection bias related to the original eligibility criteria and measurement bias due to reliance on pre-specified baseline assessments. Second, the findings may not be generalizable as they are based on an extremely small convenience sample from a specific region. Furthermore, owing to the small sample size, we could not account for or control heterogeneity within racial groups (ethnicity). Third, the presence of unknown or unmeasured confounding factors, such as the use of nonsteroidal anti-inflammatory drugs, comorbid conditions, psychosocial factors like depression or anxiety, and synovial inflammation, cannot be ruled out. Fourth, the cross-sectional design hindered our ability to determine the directionality of the relationships between variables. Finally, our study was limited by the number of biomarkers assayed.

### Conclusions

This pilot study provides pioneering evidence of race-specific relationships between inflammatory markers and knee OA symptoms among non-Hispanic Whites and Asian Americans. Based on our findings, racial/ethnic differences in this context warrant further exploration, with potential implications for the formulation of personalized strategies for managing knee OA symptoms.

## Abbreviations

CRP: C-reactive protein
IL-10: interleukin-10
KL: Kellgren–Lawrence
OA: osteoarthritis
PANAS: Positive and Negative Affect Schedule
PCS: Pain Catastrophizing Scale
SE: standard error
TNF-α: tumor necrosis factor-alpha
WOMAC: Western Ontario and McMaster Universities Osteoarthritis Index
